# Implication of the gut microbiome composition of type 2 diabetic patients from northern China

**DOI:** 10.1038/s41598-020-62224-3

**Published:** 2020-03-25

**Authors:** Qian Li, Yujun Chang, Ke Zhang, Hao Chen, Shiheng Tao, Zhi Zhang

**Affiliations:** 1 College of Life Science, State Key Laboratory of Crop Stress Biology for Arid Areas, Northwest A&F University, Yangling, Shaanxi China; 2 Bioinformatics Center, Northwest A&F University, Yangling, Shaanxi China; 3 Department of Biomedical Engineering, Medical Systems Biology Research Center, Tsinghua University School of Medicine, Beijing, China; 4 National Engineering Research Center for Beijing Biochip Technology, Beijing, China; 5 Department of Emergency Medicine, The First Affiliated Hospital, Zhengzhou University, Zhengzhou, China; 6 CapitalBio Corporation, Beijing, China

**Keywords:** Metagenomics, Type 2 diabetes

## Abstract

Emerging evidence has suggested the association of the gut microbiome with some human diseases, including type 2 diabetes (T2D). In this study, we analyzed the gut microbiota from a cohort of healthy and diabetic Chinese individuals from Northern China. Pyrosequencing of the V4V5 region of 16S rRNA genes revealed a significant decrease in the gut microbiota diversity of diabetic patients as compared to healthy individuals. Butyrate-producing bacteria such as *Bifidobacterium* and *Akkermansia* were significantly decreased in diabetic patients. Furthermore, the abundance of *Dorea* was significantly increased in T2D individuals and negatively correlated with the abundance of butyrate-producing bacteria. The increase of *Dorea* could play a role in the development of T2D and has been previously overlooked. Importantly, functional analysis of the gut microbiome revealed for the first time that increased levels of butyrate production via transferases and the degradation of several amino acids due to gut microbial metabolism have strong correlations with T2D in Northern China. Moreover, the potential of gut microbiota-based classifiers to identify individuals with a high risk for T2D has been demonstrated in this study. Taken together, our findings have revealed a previously unappreciated association of the gut microbiome with T2D and have also suggested that changes in gut microbiota may be used to identify individuals at high risk for T2D.

## Introduction

Type 2 diabetes (T2D), which is a major risk factor for heart disease and stroke, has become the leading disease burden worldwide^1^. Over the past decades, the incidence of T2D has been increasing in many industrialized countries in Europe and North America^1,2^. Currently, T2D has also sharply increased in Asian countries, in particular in China^3,4^. T2D is a metabolic disease, and the development of T2D results mostly from obesity-linked insulin resistance^5^. A previous study has indicated that as a chronic disease T2D is also associated with other factors such as gut microbiota, genetic predisposition, physical inactivity and mental stress^6^.

Recent studies have provided evidence that the human gut microbiota is critical for maintaining physical health and is related to diabetes and other disease conditions^7–9^. Previous research has focused on fecal microbiota, using primarily 16S rRNA and whole-genome shotgun sequencing, and has provided evidence that both the composition and function of gut microbial communities were critical for maintaining physical health^10^. Additionally, it has also been associated with metabolic diseases like diabetes and obesity^9^. For example, several studies on humans have indicated that a lower proportion of *Bacteroidetes* and a higher proportion of *Firmicutes* were associated with obesity and insulin resistance^9,11–13^. However, results conflicting this were reported by Larsen *et al*.; they proposed that the ratio of *Bacteroidetes* to *Firmicutes* correlated positively and significantly with plasma glucose concentrations, and the class *Betaproteobacteria* was highly enriched in diabetic individuals’ gut microbiome^8^. Consistent with these results, Ridaura *et al*. demonstrated that *Bacteroidetes* drive the degradation of branched-chain amino acids, which was reported in obese and insulin-resistant humans^14^. Yet all of these studies have confirmed the critical role of human gut microbiota in the occurrence of T2D and the maintenance of physical health.

Potential differences in gut microbiota composition related to diabetes may result in markers that can be used for disease monitoring. To date, specific gene markers and gene clusters have been used to classify T2D individuals^7,9^. Karlsson *et al*. found that metagenomic profiles could be used to identify T2D with high accuracy from a European women cohort^7^. They also applied their model to a Chinese cohort, and they found that their model was able to distinguish T2D patients and healthy adults as defined by gene clusters (MGCs) with an area under the receiver operating characteristic curve (AUC) of 0.58 for Chinese T2D subjects^7^. However, the most discriminatory MGCs differed between the European and Chinese cohorts, indicating that T2D metagenomic predictive tools and diagnostic biomarkers for specific populations need to be further studied. Moreover, 16S rRNA sequencing might be a more cost-effective method for microbiota characterization than the whole-genome shotgun sequencing and studies using the fecal microbial community structure (i.e., combinations of OTUs) to predict diabetes in adults are lacking.

In this study, we compared the fecal microbiota of T2D patients and healthy controls (n = 60). The aim of this study was to characterize the composition of gut microbial communities in adults with T2D. Furthermore, we examined whether gut microbial communities could be used to predict the presence of type 2 diabetes. We found significant shifts in the gut microbiota of patients with T2D, and we further investigated the potential use of gut microbiota profiling to accurately differentiate T2D patients from adults without diabetes.

## Results

### Diversity of the gut microbiota in patients with T2D

A total of 60 subjects were recruited to this study. The mean ± SD BMI and fasting blood glucose (FBG) levels in the T2D subjects were 26.57 ± 1.99 kg/m^2^ (control: 21.01 ± 1.51 kg/m^2^) and 7.27 ± 1.39 mmol/L (control: 5.23 ± 1.04), respectively (Supplementary Tables S1, S2). A total of 7,548,898 reads were obtained for 58 subjects by V4V5 16S rRNA pyrosequencing, and two subjects were excluded due to technical problems in sequencing. After quality control and pair-end read merging, we obtained 6,153,916 high quality reads, accounting for 81.52% of the total reads. An average of 106,102 (from 31,016 to 245,873) sequences per sample was used for downstream bioinformatics analysis, and the average sequence length of the merged sequences was 389 bp.

**Figure 1:**
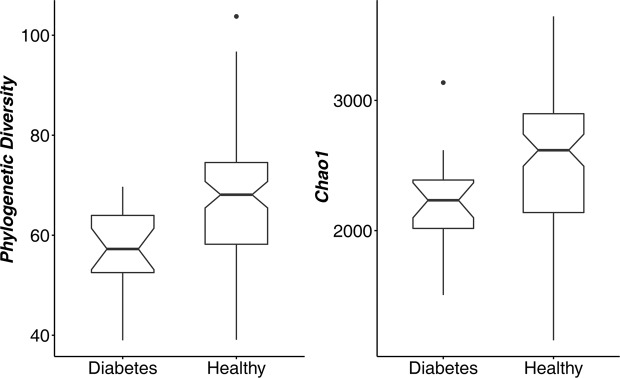
Difference in alpha diversity of the microbial communities between type 2 diabetes (T2D) and healthy individuals. (**a**) Phylogenetic diversity analysis between microbial genera. (**b**) Diversity analysis based on the Chao1 index revealing the decrease of microbial diversity in T2D. Boxes represent the interquartile ranges (IQRs) between the first and third quartiles, and the line inside the boxes represents the median; notches show the 95% confidence interval for the medians. *P*-values were < 0.01 for both phylogenetic diversity and Chao1 indices.

To estimate the diversity of the microbial communities, phylogenetic diversity and Chao1 indices were calculated and used for further comparison of the differences between the healthy and T2D groups. The individual samples were normalized, and an OTU table within each sample was rarefied to 30,000 sequence reads by QIIME 1.9.0 scripts^15^. After that, the phylogenetic diversity, Chao1 and coverage were calculated using the normalized sequence reads. Good’s coverage was estimated to be 98.05% for T2D patients and 97.78% for healthy controls. Both the phylogenetic diversity and Chao1 indices were significantly different between the two groups (Fig. 1, *p*-value = 0.0004 and 0.005 for phylogenetic diversity and Chao1 indices, respectively, Supplementary Fig. S2).

### Changes in gut microbiota

**Figure 2:**
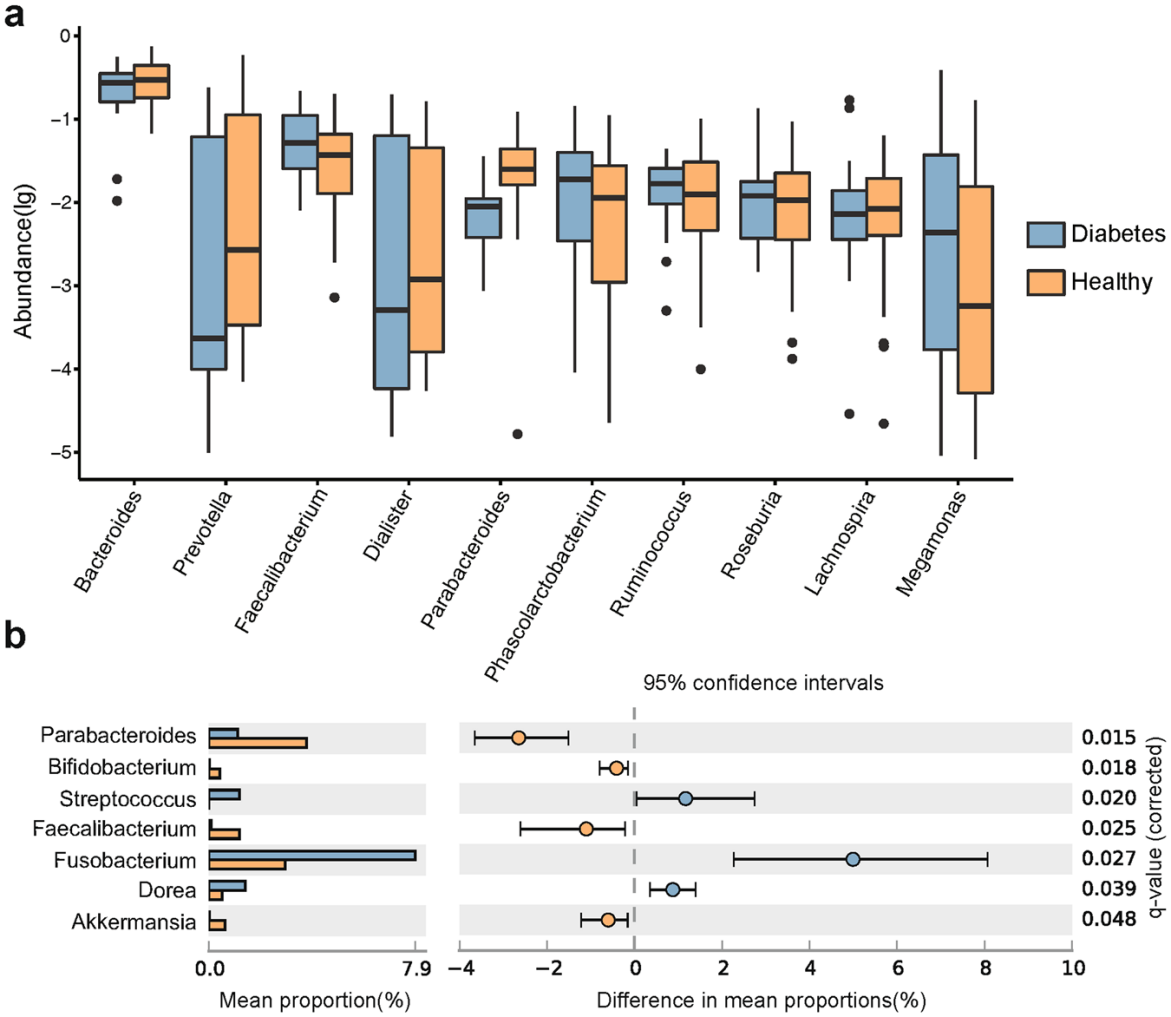
Bacterial taxonomic analysis of gut microbiota. (**a**) Boxplot showing the top 10 gut bacteria of taxonomic abundance in the two groups at the genus level. Red and blue boxes represent type 2 diabetes (T2D) and healthy controls, respectively. (**b**) Seven bacteria at the genus level that were differentially abundant between T2D patients and healthy controls, as tested by a two-sided White’s non-parametric *t*-test. FDR-adjusted *p* values are reported at the right of the image. Figure was produced using STAMP.

Taxon-based analysis revealed that the gut microbial communities were changed by diabetes at the phylum and genus level. The representative sequences of OTUs were aligned against the Greengenes database, and we summarized the taxonomic composition for all samples at the taxonomic levels of phylum, class, order, family and genus. We observed that OTUs belonging to the genus *Bacteroides* were the dominant bacteria in both groups (Fig. 2a). We then performed the two-sided White’s non-parametric *t*-*test* to identify differences in the gut microbiome between T2D and healthy groups^16^. Consistent with previous studies, at the phylum level, an increase in *Firmicutes* abundance and a relatively lower abundance of *Bacteroidetes* were found in diabetic subjects (*q*-value < 0.05)^8,9^. Obesity-related research also has revealed that obesity is associated with an increase in the phylum *Firmicutes* and a decrease in the phylum *Bacteroidetes*^11,17,18^. The relative abundances of some microbes between these two groups at the genus level were also different (Fig. 2b). At the genus level, *Faecalibacterium*, primarily presented by the species *Faecalibacterium prausnitzi* (phylum Firmicutes class Clostridia), was on average slightly increased in diabetic subjects (White’s non-parametric test, *p* = 0.015). Similarly, the proportion of the genus *Dorea*, which also belongs to the phylum *Firmicutes*, was significantly higher in diabetics than in controls (*p* = 0.038). Another genus *Fusobacterium*, belonging to the phylum *Fusobacteria*, also increased in diabetics to a significant level (*p* = 0.019). However, the relative abundance of the genus *Parabacteroides*, belonging to the phylum *Bacteroidetes*, was significantly lower in diabetics than in controls (*p* = 0.012). In addition, other genera including *Streptococcus*, *Bifidobacterium* and *Akkermansia* were also increased in healthy subjects to significant levels (Fig. 2b).

The relative abundance table of OTUs was then used for principal coordinate analysis (PCoA). PCoA analysis revealed that the gut microbiota of T2D subjects showed deviation from the control group (Supplementary Fig. S3). To compare the overall gut microbiota composition between the T2D patients and controls, we performed PERMANOVA analyses (permutational multivariate analysis of variance) with 999 permutations and confirmed significant differences between the gut microbiota composition of the two groups (*p* = 0.009). The PERMANOVA results clearly showed that diabetes was a significant factor for explaining the variation in gut microbiota.

### Bacterial interaction network

**Figure 3:**
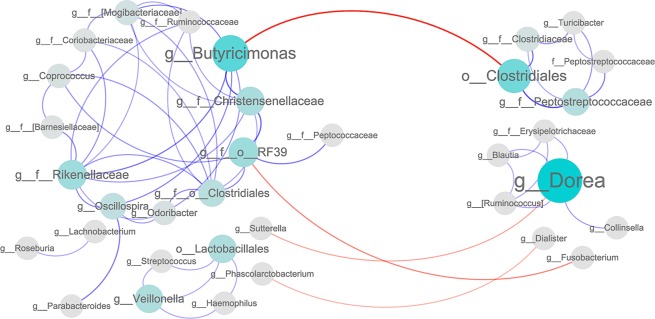
Interconnection of the type 2 diabetes (T2D) associated gut bacteria. A co-occurrence network deduced from 68 bacteria enriched in T2D subjects and controls. Nodes depict OTUs with their taxonomic assignment. The prefixed “k__,” “p__,” “c__,” “o__,” “f__,” and “g__” indicate OTUs only annotated to the level of kingdom, phylum, class, order, family or genus. Sizes of the nodes represent the relative abundance of the OTUs. Connecting lines represent Spearman’s rank correlation coefficient *p* > 0.6 (blue line) or <−0.6 (red line). The width of the connecting lines is proportional to the absolute value of the correlation coefficient.

To investigate the interactions of different gut bacteria, we performed a coabundance network analysis. This coabundance network analysis showed that OTUs annotated to *Butyricimonas* at the genus level, which have been reported to counteract T2D, were positively associated with OTUs belonging to *Rikenellaceae* and *Christensenellaceae* at the family level (Fig. 3). Another interesting finding was the presence of a few negative connections, such as between *Butyricimonas* and *Clostridiales* at the order level. In this result, *Dorea* showed a negative correlation with *Sutterella*, and *Dialister* was negatively correlated with *Phascolarctobacterium* at the genus level. These data identified various relationships between T2D-associated bacteria and suggested it may be important to further identify the roles of gut bacteria and how they interact with each other and their host in T2D.

### Functional changes in gut microbiome

We next performed PICRUSt2 analysis, which is a computational approach that predicts the metagenome functional content based on microbial community profiles obtained from 16S rRNA gene sequences, to reveal the functional differences between the two groups^19^. Statistical differences in Kyoto Encyclopedia of Genes and Genome orthology (KO) frequencies were determined using the Mann-Whitney U test. The KO assignments for the five microbial proteins with the lowest false discovery rate (FDR) adjusted p values (*p* < 0.001) were putrescine oxidase [EC:1.4.3.10] (puo), streptogrisin C [EC:3.4.21.-] (sprC), 3-hydroxyanthranilate 3,4-dioxygenase [EC:1.13.11.6] (HAAO), glycine betaine catabolism A (gbcA), phenol 2-monooxygenase [EC:1.14.13.7], and S-adenosylmethionine-diacylgycerolhomoserine-N-methlytransferase (btaB). sPLS-DA analysis was then performed to identify key genes that were important for separating diabetic and healthy individuals (Supplementary Fig. S1). These key genes were closely related to the four proteins determined by the previous Mann-Whitney U test, puo, sprC, HAAO and btaB. We then inferred gut metabolic modules (GMMs) associated with diabetes based on the KO frequencies using the online tool GOmixer^20^. A comparison of healthy controls and T2D patients showed that 11 GMMs had significant differences according to their adjusted p values (*p* < 0.05). All identified GMMs are shown in Supplementary Table S3, including tyrosine degradation, pentose phosphate, lactose and galactose degradation and butyrate production via transferase. These results suggested that the levels of tyrosine and butyrate production may be altered in individuals with T2D from northern China.

### A metagenomic classifier for T2D

**Figure 4:**
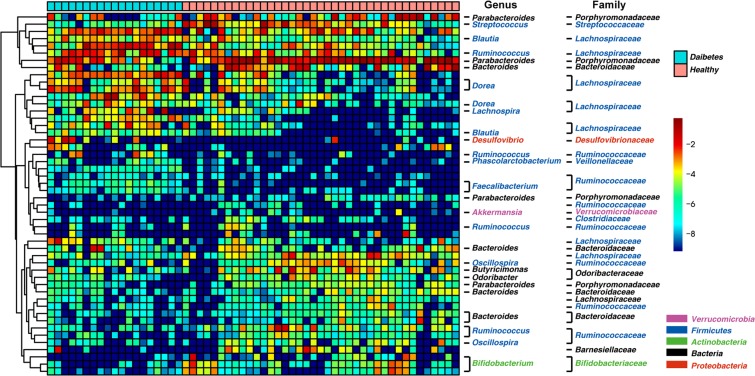
Heatmap showing the relative abundances of the 50 most predictive OTUs according to T2D classification. The color of the spot corresponds to the log-transformed relative abundance of the OTU. The genus names of the OTUs are labeled on the right.

To exploit the suitability of the gut microbiome for T2D classification, we evaluated the predictive power of the gut microbiota taxonomic community composition using random forest (RF) analysis. First, we removed OTUs that were rare and found in less than 20% of the samples because these OTUs were less likely to help in model construction. Subsequently, the microbiome data were transformed via an inverse hyperbolic sine and then to mean center by sample^21^. The RF classification model was used based on the standardized data after these transformations (for the selection of the most discriminatory OTUs between the two groups). According to the importance score obtained from this RF analysis, the 50 most discriminatory OTUs were selected as depicted in Fig. 4. Several OTUs from *Bifidobacterium*, *Parabacteroides*, *Oscillospira*, and *Bacteroides* and one OTU from family *Lachnospiraceae* were associated with healthy samples. Other OTUs belonging to members of *Faecalibacterium*, *Dorea*, *Clostridiales*, and *Clostridiaceae* and another OTU from family *Lachnospiraceae* were associated with T2D samples.

**Figure 5:**
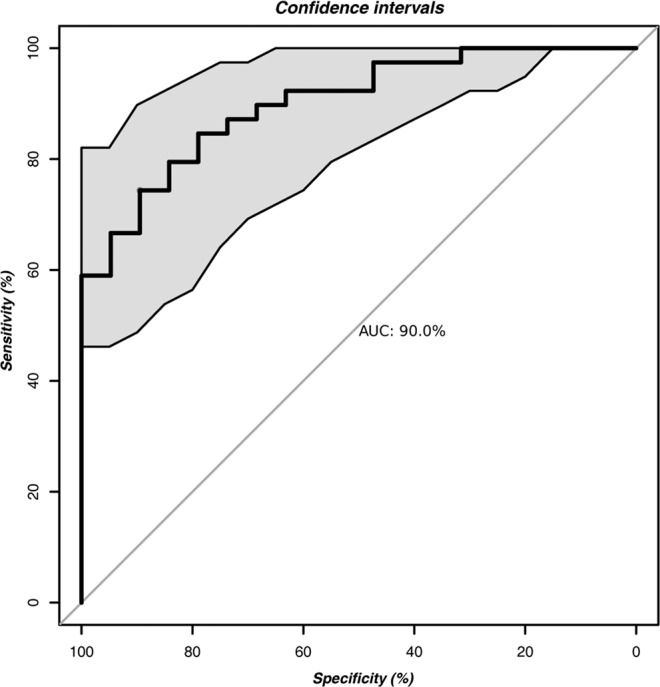
The area under the ROC curve (AUC) of gut-microbiota-based T2D classification. Random forest classifiers were used to separate T2D patients and healthy controls based on the OTU-level gut microbiome composition. The grey area between the two outside curves represents the 95% confidence interval (CI) shape.

After discriminatory OTUs were selected, a second RF classifier was trained based on the 50 most discriminatory OTUs. The performance of this RF classification model based on the most discriminatory OTUs was quantified by an area under the receiver operating characteristic (ROC) curve (AUC) of 0.90 for the validation set, corresponding to a specificity of 0.89 and a sensitivity of 0.74 (Fig. 5). Overall, in this assessment analysis, we demonstrated the discriminatory power of our classifiers based on cross-validation.

## Discussion

Evidence that the gut microbiota is associated with the development of T2D is rapidly accumulating. Thus, the characterization of the gut microbiota in diabetes and the identification of gut metagenomic markers that can differentiate T2D cases and controls is important. In this study, we reported significant differences in the gut microbial composition of individuals with T2D as compared to a healthy cohort. Moreover, we combined univariate analysis methods and supervised classification methods to finally identify several bacterial genera that were differentially abundant between the microbiota of diabetes and non-diabetic controls. Most previous studies have only considered unsupervised classification methods. Additionally, to our knowledge, only few related studies based on the gut microbiota of China gut individuals have been published thus far^8,9^. These findings have all supplemented worldwide gut microbiota research related to T2D.

We identified that gut microbe diversity was significantly decreased in T2D patients consistent with previous studies in different populations of the world, including other populations in China^8,22,23^. The complex interactions of intestinal microbiota with the gut mucosa could play a key role in the pathogenesis of T2D, which is similar to other diseases such as obesity and inflammatory bowel disease^24–26^. The decrease in the gut microbe diversity of T2D patients could induce a certain degree of gut bacterial dysbiosis and interfere with the interaction between gut microbiota and hosts. For example, our results showed that at the genus-level, alterations in the gut microbiome in T2D samples had certain patterns. Butyrate-producing bacteria, such as those of the *Faecalibacterium*, *Bifidobacterium*, and *Akkermansia* genera^14,27,28^, decreased in diabetic persons. Conversely, some bacteria that can cause chronic inflammation increased^24^.

After having analyzed the diversity and composition of the gut microbiota and the microbial features associated with T2D, we addressed the functional features of gut microbiota. Taking the PICRUSt2 predictions and GMMs annotations together, we identified that the metabolism of several amino acids, such as tyrosine and alanine, was associated with the development of T2D. Specifically, we demonstrated that in comparison to healthy controls, the overexpression of microbial proteins such as sprC, HAAO, and gbcA has a significant correlation with the insulin resistance of T2D individuals. The gut mucosal barrier is critical for increasing insulin sensitivity and preventing the development of diabetes^29^. However, sprC is involved in cell motility, and may play an important role in the process of bacteria penetrating the mucus lining of the colon^29,30^. HAAO may participate in tryptophan metabolism and the synthesis of quinolinic acid leading to inflammatory disorders and insulin resistance^31,32^. gbcA in bacteria is related to glycine betaine which is an intermediate in the catabolism of choline and carnitine^33^. As a result, it might interact with glycine metabolism in humans, leading to insulin resistance and an increased risk of T2D^34^. However, the relationship between the metabolism of gut microbiota and hosts with T2D is complex, and further animal experimental and metabolomics studies will be required to clarify the mechanisms of regulation of metabolites by the gut microbiota. In summary, these findings highlight the possibility that the alteration of the gut microbial composition in T2D patients could destroy the gut microbiota balance, leading to functional dysbiosis and an increase in the susceptibility of a host to diabetes. Furthermore, modulation of tyrosine metabolism and butyrate production may be a potential method for improved prevention of type 2 diabetes.

We also validated the discriminatory power of our selected gut microbial markers using supervised learning techniques. When applying a random forest model, we found that our predictive model with a combination of 50 OTUs was able to distinguish T2D patients with a sensitivity of 0.74 and specificity of 0.90. Our results support the current viewpoint that gut microbiota-based classifier, especially using 16S rRNA sequencing technology, could be used to discriminate T2D individuals from healthy individuals. Furthermore, a random forest model could also be utilized to identify the bacterial taxa associated with disease activity. Overall, it is worthwhile to identify potential individuals that are at high risk for T2D, but more validation of the performance of gut microbiota based classifiers in T2D patients in other populations across the world is required.

To interpret our findings on T2D gut microbiota further based on individuals from northern China, we compared them with 50 samples from America. We have downloaded raw data of 50 samples from the official HMP project database containing 20 diabetic and 30 healthy samples as representatives of an American cohort. PCoA and PERMANOVA analyses showed significant differences between the cohort in our study and the American cohort, both in terms of the diabetes group and the healthy group (*p* < 0.05) (Supplementary Fig. S4). According to the results of this comparative analysis, we therefore conclude that the cohort in this study was at least specific to China, consistent with the previous findings of Karlsson *et al*.^7^.

In conclusion, our findings add extra insight to the association between the gut microbiota and diabetes. Moreover, our analysis suggests an association of microbial tyrosine metabolism in the gut is related to diabetes. We have also validated the discriminatory power of a gut microbiota-based T2D classier in populations from northern China. However, longitudinal studies using detailed information about the interaction between the proteins or metabolites of gut microbiota and host-associated diabetes progression are still needed.

## Methods

### Study population and sample collection

Forty healthy subjects and twenty newly diagnosed T2D subjects were recruited for this study and signed informed consent. All healthy subjects were recruited to this study after physical examination and health assessment. All T2D subjects were newly diagnosed and did not previously receive any treatment or medication. T2D subjects were required to meet the following inclusion criteria: (i) fasting blood glucose test (FBG) 7 mmol/L or greater and/or 2-h fasting oral glucose tolerance test (OGTT) 11.1 mmol/L or greater^35^; (ii) no previously received pharmacologic treatment; and (iii) body mass index (BMI) > 18.0 kg/m^2^. To eliminate the effects of other factors on the gut microbiota, we conducted additional questionnaire survey of all subjects and excluded individuals according to certain criteria: (i) age less than 20 or greater than 60; (ii) antibiotic usage within two months; (iii) habitual probiotic or yogurt consumption; (iv) tobacco or alcohol abuse. In addition, we also excluded individuals in T2D group based on the following criteria to eliminate the effects of other diseases on gut microbiota: (i) gastrointestinal related diseases or infection within the previous two months; (ii) clinically significant major systemic diseases, including cancer and autoimmune diseases; and (iii) cardiovascular- or cerebrovascular-related diseases. BMIs were calculated using the formula: weight (kg)/height (m^2^). Fecal samples were frozen immediately in a −80 °C freezer after collection. After recruitment to the study, one T2D subject and one non-diabetic subject were excluded due to technical problems with sequencing.

### Fecal processing and pyrosequencing

Genomic DNA was extracted using a QIAamp DNA stool mini kit (Qiagen, Hilden, Germany), and the amount of extracted DNA was checked using a Qubit 2.0 Fluorometer (Life Technologies, USA). The extracted genomic DNA was used to construct an amplicon library by amplifying the V4V5 region of the 16S rRNA gene. The PCR reaction was performed on a thermocycler and the PCR amplicons were sequenced using an Illumina Miseq according to the manufacturer’s protocol. A negative control sample (PCR-grade Water) was included in DNA extraction and handled identically to the faecal samples to control for DNA contamination.

### 16S rRNA gene sequence analysis

The raw sequencing reads from the Illumina Miseq Platform were first treated using Trimmomatic v0.36 to reduce error rates^36^. Sequence adapters and low-quality bases from paired reads were trimmed or filtered. Paired-end reads were then merged by fastq-join if there were at least 10 bases of overlap^37^. Merged sequences were clustered using the uclust algorithm into operational taxonomic units (OTUs) with a threshold similarity of 97% against the Greengenes reference and taxonomy version 13_8 was used^38,39^.

Rarefaction analysis was performed based on the representative sequences for shared OTUs. In addition, community richness diversity was compared between the two groups based on the phylogenetic diversity index and Chao1 values were calculated by QIIME 1.9^15^. An OTU-to-OTU co-occurrence network was built using Cytoscape v3.6.0 based on Spearman’s rank correlation coefficient^40,41^. In the co-occurrence network analysis, only OTUs present in at least 20% samples were used, and only connections with a *rho* larger than 0.6 or smaller than −0.6 were used for network building (*p*-value < 0.01). In order to compare the community structure of the two sample groups, the significance values were computed based on permutational multivariate analysis of variance (PERMANOVA) with Bray-Curtis dissimilarity^42^. Each representative sequence was assigned to bacterial taxa by a Ribosomal Database Project (RDP) classifier, and the relative abundances of the bacterial taxa at the phylum, genus, and species level were calculated^43^. For functional metagenome analysis, we reconstructed the metagenome using PICRUSt2 based on the OTU table^44^. All predicted functional genes were categorized into Kyoto Encyclopedia of Genes and Genome Orthology (KO). Supervised sparse partial least squares discriminant analysis (sPLS-DA) was also used to identify genes that may discriminate the two groups through the mixOmics R package^45^. KOs were assigned to gut metabolic modules (GMMs) and evaluated using GOmixer^20^.

### Statistical analysis

We performed a differential abundance analysis at the genus and OTU level and the White’s non-parametric t-test was performed to determine whether the difference was statistically significant^46^. In order to control the false discovery rate, the adjusted *p*-values were computed by applying the popular FDR algorithm^47,48^. The random forest model has been shown to be a suitable model for exploiting microbiome data^49^. In the current study, only OTUs present in at least 80% of samples were used for further analyses. Random forest models were trained using the filtered profiles of OTUs and genus to identify diabetes patients in test sets of diabetic and non-diabetic subjects using the random forest package in R^46^. The performance of this predictive model was then evaluated with a fivefold cross-validation approach and measured by the receiver operating characteristic (ROC) curve and the area under the ROC curve (AUC). Prior to the random forest analysis, the microbiome data were transformed via an inverse hyperbolic sine transformation and then mean centered per subjects^21^. The variable importance by mean decrease in accuracy was calculated to find the most discriminatory OTUs between the diabetic and non-diabetic individuals. The smaller random forest model was trained containing only the 50 most discriminatory OTUs to classify diabetic patients from subjects in R with default parameters and 500 trees.

### Ethics statement

All individuals included in the present study gave written informed consent before participation in the study. The study was approved by the Ethical Committees of the Tsinghua University and performed according to the declaration of Helsinki.

## Supplementary information

Supplementary Information.

## Data Availability

The datasets generated during the current study have been deposited in the EBI Sequence Read Archive under accession number ERP107659.
