# Supplementary material for: Implication of the gut microbiome composition of type 2 diabetic patients from northern China
Source: Sci Rep. 2020 Mar 25;10:5450. doi: 10.1038/s41598-020-62224-3 (PMC7096501; doi:10.1038/s41598-020-62224-3)
Supplement: Supplementary file 1 — Supplementary Information. [file 41598_2020_62224_MOESM1_ESM.pdf]

Implication of the gut microbiome composition of type 2 diabetic patients from northern China

Qian Li[1,2,#], Yujun Chang[3,4,6,#], Ke Zhang[4,6], Hao Chen[5], Shiheng Tao[1,2,*], Zhi Zhang[4,6 *]

[1] College of Life Science, State Key Laboratory of Crop Stress Biology for Arid Areas, Northwest A&F University, Yangling, Shaanxi, China

[2] Bioinformatics Center, Northwest A&F University, Yangling, Shaanxi, China

[3] Department of Biomedical Engineering, Medical Systems Biology Research Center, Tsinghua University School of Medicine, Beijing, China

[4] National Engineering Research Center for Beijing Biochip Technology, Beijing, China

[5] Department of Emergency Medicine, The First Affiliated Hospital, Zhengzhou University, Zhengzhou, China

[6] CapitalBio Corporation, Beijing, China

[#] **Equal contribution**

*** Correspondence:**
Shiheng Tao, Zhi Zhang

* Correspondence to chang349276@126.com shihengt@nwafu.edu.cn

Supplementary Figure S1.

sPLS-DA analysis of microbial proteins based on KO frequencies. The gut microbial proteins distinguishes individuals with T2D from control subjects. a: sPLS-DA of microbial proteins comparing healthy control and diabetes groups. Yellow triangle, healthy individuals; blue circle, individuals with diabetes. b: Component 1 contributing variables.

a

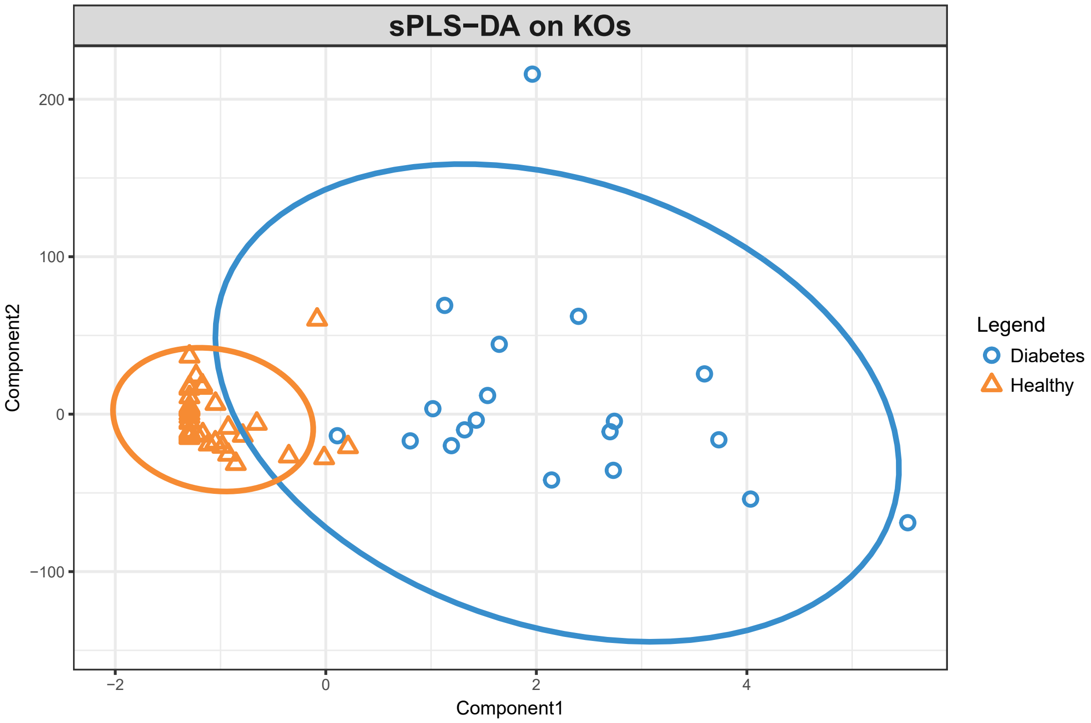

b

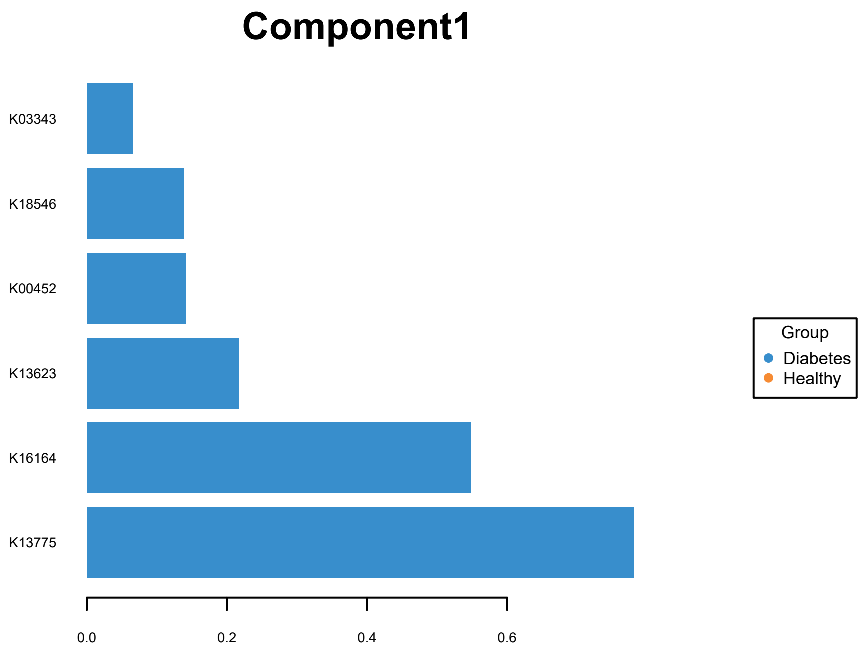

Supplementary Figure S2.

Alpha diversity of the microbial communities between T2D and healthy individuals. (a) Shannon index ($p = 0.54$); (b) Observed species ($p = 0.0049$); (c) Good's coverage ($p = 0.0023$); (d) Simpson index ($p = 0.37$). Boxes represent the interquartile ranges (IQRs) between the first and third quartiles, and the line inside the box represents the median; notches show the 95% confidence interval for the medians.

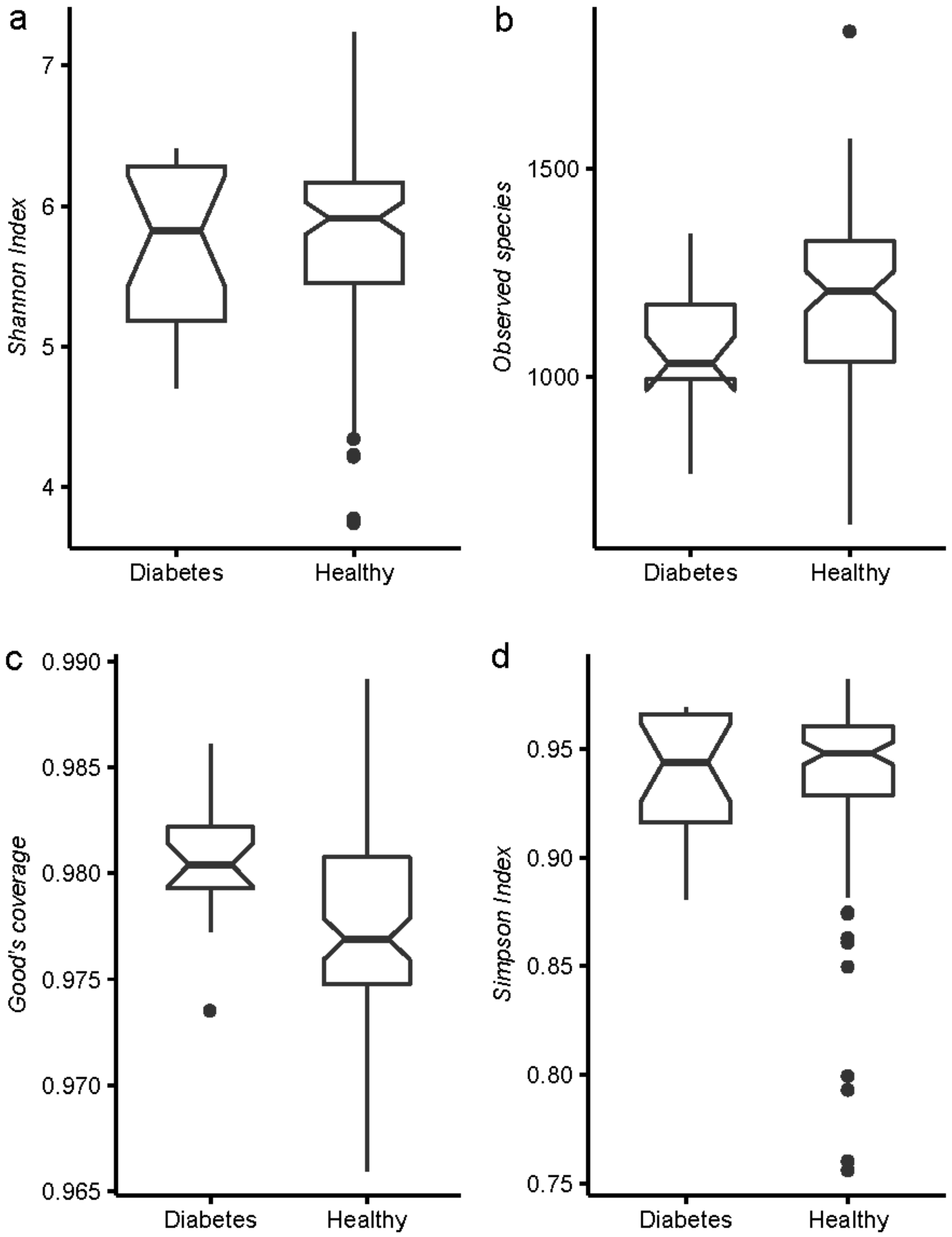

Supplementary Figure S3.

PCoA of gut microbiota based on the OTU data. Red circles represent subjects with T2D, green triangles represent subjects in the healthy group.

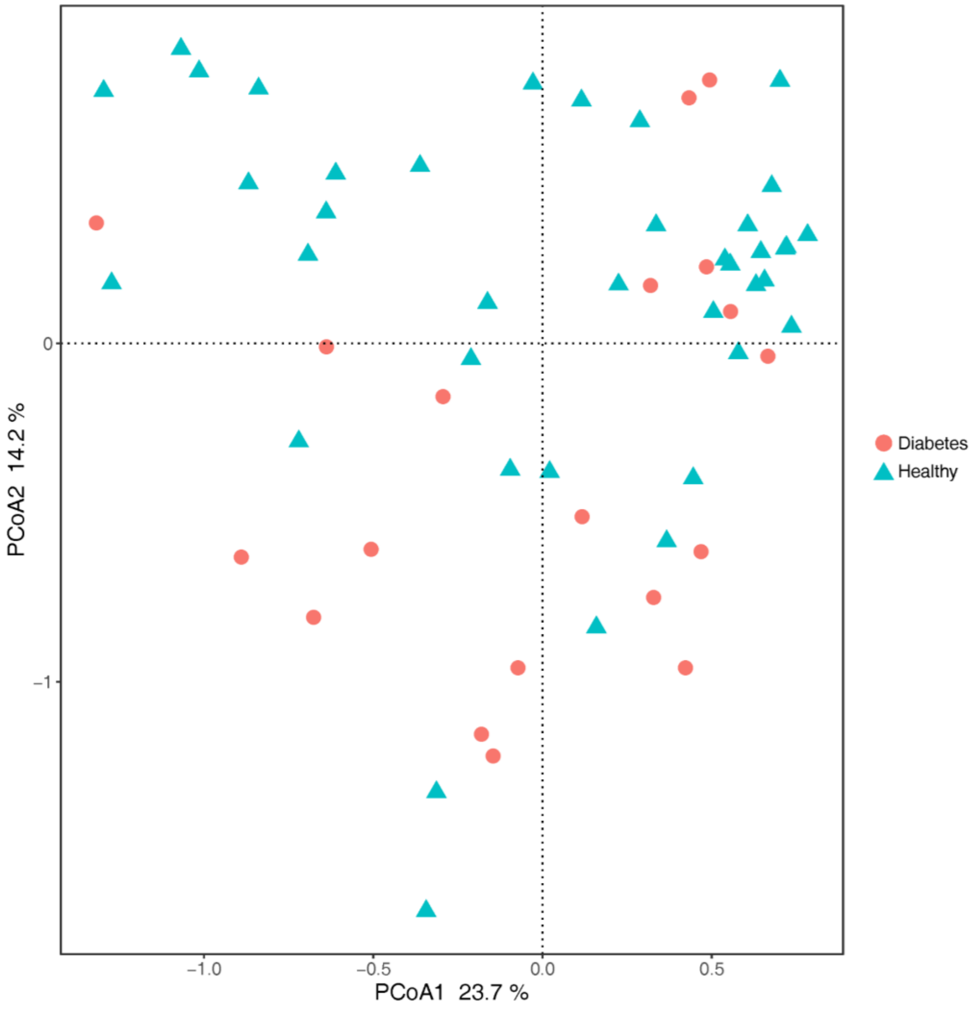

Supplementary Figure S4.

PCoA of gut microbiota based on the OTU data for samples in our study and HMP. (a) PCoA of gut microbiota showed clear differences between diabetic samples in our study and HMP (PERMANOVA, $p < 0.05$). Blue circles represent T2D subjects in our study, green triangles represent T2D subjects in HMP. (b) PCoA of gut microbiota showed clear differences between healthy samples in our study and HMP (PERMANOVA, $p < 0.05$). Red circles represent healthy subjects in our study, cyan triangles represent healthy subjects in HMP.

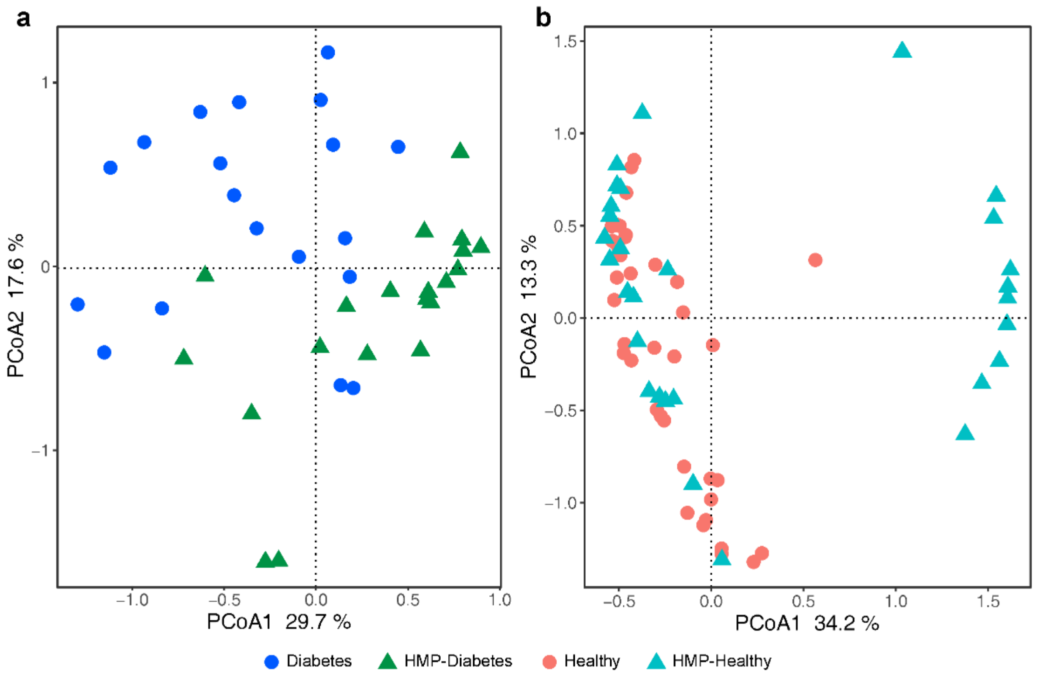

Table S1 Summary of clinical characteristics

Variable	Control	Type 2 diabetic subjects
N	40	20
BMI (kg/m^2)	21.01 ± 1.51	26.57 ± 1.99
FBG (mmol/L)	5.23 ± 1.04	7.27 ± 1.39
2hOGTT (mmol/L)	8.23 ± 0.88	13.13 ± 3.54
TC (mmol/L)	4.40 ± 0.80	5.28 ± 0.99
TG (mmol/L)	1.10 ± 0.27	1.79 ± 0.91
HDL (mmol/L)	1.42 ± 0.21	1.28 ± 0.22
LDL (mmol/L)	2.54 ± 0.49	2.70 ± 0.47

Data are expressed as the mean ± SD. BMI, body mass index; FBG, fasting blood glucose; 2hOGTT, 2-h fasting oral glucose tolerance test; TC, total cholesterol; TG, triglycerides; HDL, high-density lipoprotein cholesterol; LDL, low-density lipoprotein cholesterol.

Table S2 Clinical characteristics of all subjects

SampleID	Sex	BMI	FBG	2hOGTT	TC	TG	HDL	LDL	Group
CDTC160057	male	26.8	8.27	15.71	5.94	1.09	1.43	3.12	T2D
CDTC160058	male	26	11.47	22.95	4.65	0.83	1.36	2.36	T2D
CDTC160059	male	25.8	7.94	16.47	5.9	3.92	1.31	2.84	T2D
CDTC160060	female	29.1	8.27	15.48	6.25	2.22	1.5	3.04	T2D
CDTC160061	female	29.7	7.34	10.84	5.31	1.52	1.26	2.66	T2D
CDTC160062	female	24.6	4.89	11.47	4.26	0.56	1.5	1.97	T2D
CDTC160063	male	25.2	7.6	14.58	5.71	2.29	1.29	2.86	T2D
CDTC160064	female	25.2	7.2	12.68	6.16	1.24	1.35	3.13	T2D
CDTC160065	female	31.3	6.48	12.95	6.93	2.15	1.66	3.38	T2D
CDTC160066	male	27.9	7.75	6.39	7.09	3.43	1.59	3.41	T2D
CDTC160067	male	25.8	7.11	9.45	4.41	0.91	1.23	2.22	T2D
CDTC160068	male	28.9	7.64	17.11	5.86	2.82	1.09	3.15	T2D
CDTC160069	male	27.1	6.02	11.35	6.23	1.23	1.39	3.33	T2D
CDTC160070	male	25.2	4.79	12.96	4.41	2.14	1.1	2.22	T2D
CDTC160071	female	27	7.42	10.1	3.76	1.04	1.11	1.91	T2D
CDTC160072	male	25.4	6.41	13.55	4.94	1.32	1.36	2.7	T2D
CDTC160073	male	25	8.13	9.76	4.29	1.46	0.97	2.3	T2D
CDTC160074	female	27.3	6.44	11.78	4.96	2.82	1.04	2.67	T2D
CDTC160075	female	23.7	7.27	15.43	4.22	1.35	0.96	2.3	T2D
CDTC160076	female	24.3	6.9	11.5	4.31	1.45	1.02	2.4	T2D
CDTC160039	female	20.7	3.63	7.72	2.93	1.42	1.36	2.29	Healthy
CDTC160040	female	23.81	6.56	8.49	3.98	1.06	1.69	3.02	Healthy
CDTC160041	female	20.32	6.77	10.17	4.12	1.12	1.39	2.38	Healthy
CDTC160042	female	20.2	3.53	7.53	3.88	0.74	1.35	2.36	Healthy
CDTC160043	male	20.57	3.85	7.42	2.99	1.04	1.29	1.87	Healthy
CDTC160044	female	22.32	4.96	8.58	3.9	1.12	1.47	1.92	Healthy
CDTC160045	female	22.49	5.56	8.32	5.07	0.91	1.9	2.86	Healthy
CDTC160046	male	19.03	4.4	7.53	3.91	1.19	1.54	2.42	Healthy
CDTC160047	female	18.75	4.99	8.03	4.39	0.68	1.5	2.21	Healthy

CDTC160048	female	18.73	4.31	7.36	2.97	0.8	0.98	2.89	Healthy
CDTC160049	female	23.81	6.9	10.32	3.14	1.49	1.12	3.17	Healthy
CDTC160050	female	20.31	4.94	7.54	3.67	1.4	1.75	2.7	Healthy
CDTC160051	female	22.19	7.76	9.15	4.72	1.23	1.33	2.49	Healthy
CDTC160052	male	21.55	5.05	8.12	5.16	1.16	1.24	3.09	Healthy
CDTC160053	female	22.86	5.59	8.98	5.03	1.44	1.63	2.71	Healthy
CDTC160054	female	22.89	4.22	7.79	4.63	1.12	1.56	2.76	Healthy
CDTC160055	female	20.93	3.43	7.61	3.94	1.59	1.06	2.66	Healthy
CDTC160056	male	23.67	5.65	7.23	4.53	1.26	1.53	2.95	Healthy
CDTC160004	female	22.76	5.79	8.53	5.34	1.19	1.34	2.95	Healthy
CDTC160006	female	20.07	5.59	8.22	6.24	1.49	1.49	1.41	Healthy
CDTC160007	female	20.07	4.35	6.97	3.87	0.79	1.51	2.81	Healthy
CDTC160009	female	20.7	6.53	8.48	6.51	1.17	1.47	3.31	Healthy
CDTC160010	female	19.7	5.67	8.01	3.98	0.91	1.26	2.51	Healthy
CDTC160011	female	21	5.12	7.63	4.14	0.85	1.68	3.13	Healthy
CDTC160012	female	21.29	4.33	6.86	3.72	1.14	1.49	3.07	Healthy
CDTC160014	female	21.5	4.42	7.21	4.16	0.6	1.37	2.38	Healthy
CDTC160015	female	19.5	4.74	8.56	4.85	1.39	1.24	1.72	Healthy
CDTC160016	female	21.9	6.31	9.92	4.33	1.29	1.19	2.79	Healthy
CDTC160017	female	20.54	4.8	7.82	4.47	1.18	1.67	3.01	Healthy
CDTC160020	male	19.36	5.98	8.99	4.46	0.72	1.27	2.14	Healthy
CDTC160025	female	18.99	4.77	7.79	4.96	1.05	1.05	1.73	Healthy
CDTC160027	female	19.5	5.27	9.18	4.39	0.71	1.55	2.45	Healthy
CDTC160028	female	18.8	4.81	7.91	4.08	1.34	1.25	2.69	Healthy
CDTC160029	female	23.6	6.73	8.69	5.77	1.65	1.14	3.51	Healthy
CDTC160030	female	22.3	4.89	8.06	3.92	0.59	1.44	1.56	Healthy
CDTC160034	male	20.82	6.54	8.78	4.76	1.11	1.26	2.05	Healthy
CDTC160035	male	21.53	5.44	9.51	5.11	1.04	1.45	2.25	Healthy
CDTC160036	female	19.2	3.54	7.01	4.15	1.06	1.63	2.62	Healthy
CDTC160037	female	20.28	5.78	7.63	4.79	0.98	1.46	1.99	Healthy
CDTC160038	female	21.8	5.86	9.54	5.21	1.03	1.81	2.56	Healthy

Table S3 Gut metabolic modules comparing Healthy to Diabetes

Gut metabolic module	P.value	FDR	Direction Healthy/Diabetes
MF0114: acetyl-CoA to crotonyl-CoA	1.00E-04	0.002033	-
MF0099: methanol conversion	1.00E-04	0.002033	-
MF0021: leucine degradation	1.00E-04	0.002033	+
MF0008: tyrosine degradation (hydroxyphenylacetaldehyde pathway)	1.00E-04	0.002033	+
MF0106: anaerobic fatty acid beta-oxidation	3.00E-04	0.005229	+
MF0133: menaquinone production	5.00E-04	0.007625	+
MF0090: pentose phosphate pathway (non-oxidative branch)	6.00E-04	0.008133	-
MF0079: bifidobacterium shunt	0.0013	0.014418	-
MF0047: lactose and galactose degradation (PTS)	0.0013	0.014418	-
MF0116: butyrate production via transferase	0.002	0.020333	-
MF0025: alanine degradation (glutamate pathway)	0.0029	0.027215	+
MF0022: isoleucine degradation	0.0061	0.053157	-
MF0092: kdo2-lipid A synthesis	0.011	0.073842	+
MF0061: mannose degradation	0.0115	0.073842	+
MF0059: rhamnose degradation	0.0105	0.073842	+
MF0026: cysteine biosynthesis/homocysteine degradation	0.011	0.073842	+
MF0024: methionine degradation (mercaptan pathway)	0.0095	0.073842	-
MF0119: lactate production	0.0127	0.076913	-
MF0087: TCA cycle (Mycobacterium pathway)	0.0145	0.076913	+
MF0063: fructan degradation	0.0133	0.076913	+
MF0033: cysteine degradation (mercaptopyruvate pathway)	0.0139	0.076913	+
MF0084: pyruvate:ferredoxin oxidoreductase	0.0191	0.097092	+
MF0083: pyruvate dehydrogenase complex	0.0248	0.121024	+
MF0086: TCA cycle	0.0282	0.132323	+
MF0065: pectin degradation - 5-dehydro-4-deoxy-D-glucuronate degradation	0.0306	0.138267	+
MF0035: arginine degradation (agmatine deiminase pathway)	0.0333	0.145093	+
MF0055: xylose degradation	0.0361	0.151869	+
MF0064: pectin degradation	0.0441	0.173555	+
MF0038: arginine degradation (arginine:pyruvate transaminase/4-aminobutyrate pathway)	0.0441	0.173555	0
MF0103: nitrate reduction (assimilatory)	0.0477	0.181856	+
MF0071: D-galacturonate degradation	0.0536	0.198158	+
MF0131: superoxide dismutase	0.0601	0.215653	+
MF0130: peroxidase	0.0647	0.225526	+
MF0057: alpha-D-glucose and alpha-D-glucose 1-phosphate degradation	0.0671	0.227394	+
MF0127: Succinate production	0.0722	0.238065	+
MF0006: urea degradation	0.0749	0.240468	+
MF0085: pyruvate:formate lyase	0.0895	0.259976	-
MF0073: sorbitol degradation (dehydrogenase)	0.0864	0.259976	-
MF0072: ribitol degradation	0.0895	0.259976	-
MF0067: PHB production	0.0895	0.259976	+
MF0110: glyoxylate bypass	0.0959	0.272088	+
MF0016: glycine degradation	0.1027	0.284759	+
MF0043: arginine degradation (agmatinase pathway)	0.1062	0.28792	-
MF0101: Sulfate reduction (assimilatory)	0.1174	0.30474	+
MF0076: arabitol degradation	0.1174	0.30474	+
MF0077: galactitol degradation	0.1214	0.308558	-

MF0093: homoacetogenesis	0.1338	0.313915	+
MF0078: xylitol degradation	0.1338	0.313915	+
MF0041: histidine degradation	0.1295	0.313915	+
MF0014: glutamate degradation (4-aminobutanoate pathway)	0.1295	0.313915	+
MF0045: trehalose degradation	0.1426	0.328249	-
MF0081: Glycolysis (pay-off phase)	0.1566	0.341164	+
MF0049: maltose degradation	0.1566	0.341164	-
MF0037: arginine degradation (AST/succinyltransferase pathway)	0.1566	0.341164	+
MF0009: tryptophan degradation	0.1769	0.378628	+
MF0003: acetylglucosamine degradation	0.1823	0.383459	+
MF0126: propionate production via transferase	0.1878	0.388332	-
MF0104: nitrate reduction (dissimilatory)	0.211	0.415194	+
MF0091: beta-D-glucuronide and D-glucuronate degradation	0.205	0.415194	+
MF0052: chondroitin sulfate and dermatan sulfate degradation	0.211	0.415194	+
MF0023: methionine degradation (cysteine pathway)	0.2171	0.420416	-
MF0129: catalase	0.2233	0.425666	+
MF0050: melibiose degradation	0.2297	0.431129	-
MF0125: propionate production via kinase	0.2496	0.454496	+
MF0017: alanine degradation (racemase pathway)	0.2496	0.454496	+
MF0095: NADH:ferredoxin oxidoreductase	0.2634	0.472571	+
MF0034: glutamine degradation (oxoglutarate pathway)	0.2852	0.504267	+
MF0121: propionate production (acrylate pathway)	0.3005	0.508278	-
MF0105: nitrate reduction (denitrification)	0.2928	0.508278	+
MF0070: galactonate degradation	0.3083	0.508278	+
MF0054: arabinose degradation	0.3005	0.508278	+
MF0044: glycocholate degradation	0.3083	0.508278	+
MF0030: threonine degradation (formate pathway)	0.3242	0.527365	-
MF0018: proline degradation (glutamate pathway)	0.3324	0.533589	+
MF0118: formate conversion	0.3408	0.539969	+
MF0128: Propionate conversion to succinate	0.3754	0.558295	+
MF0109: glycerol degradation (glycerol kinase pathway)	0.3844	0.558295	-
MF0100: Sulfate reduction (dissimilatory)	0.3754	0.558295	+
MF0060: ribose degradation	0.3844	0.558295	-
MF0042: 4-aminobutyrate degradation	0.3844	0.558295	+
MF0036: arginine degradation (ornithine decarboxylase pathway)	0.3754	0.558295	-
MF0002: ethanol production (CO2 pathway)	0.3754	0.558295	+
MF0032: glutamine degradation (ammonia pathway)	0.3935	0.564788	+
MF0089: Entner-Doudoroff pathway I	0.4028	0.571414	+
MF0051: sucrose degradation (Actinobacteria)	0.4122	0.578028	-
MF0122: propionate production (succinate pathway)	0.4217	0.58463	+
MF0039: lysine fermentation to acetate and butyrate (3,6-diaminohexanoate pathway)	0.4313	0.59122	-
MF0005: acetylneuraminate and acetylmannosamine degradation	0.4411	0.597936	+
MF0108: glycerol degradation (dihydroxyacetone pathway)	0.4814	0.611779	-
MF0107: glycerol degradation (propanediol pathway)	0.461	0.611779	-
MF0088: TCA cycle (Helicobacter pathway)	0.4711	0.611779	+
MF0082: pentose phosphate pathway (oxidative branch)	0.4814	0.611779	+
MF0074: mannitol degradation	0.4711	0.611779	-
MF0046: sucrose degradation	0.4814	0.611779	-
MF0115: Crotonyl-coA from succinate	0.4917	0.618427	-
MF0015: glutamate degradation (methylaspartate pathway)	0.5022	0.625188	-
MF0132: superoxide reductase	0.5344	0.658554	+
MF0111: triacylglycerol degradation	0.5676	0.678894	+

MF0094: hydrogen metabolism	0.5676	0.678894	-
MF0080: Glycolysis (preparatory phase)	0.5676	0.678894	-
MF0001: ethanol production (formate pathway)	0.5903	0.69919	-
MF0124: Fucose degradation	0.6133	0.699277	+
MF0117: butyrate production via kinase	0.6133	0.699277	-
MF0075: sorbitol degradation (phosphotransferase)/sorbose degradation	0.6133	0.699277	+
MF0004: putrescine degradation	0.6018	0.699277	-
MF0113: acetyl-CoA to acetate	0.625	0.706019	-
MF0097: Methanogenesis - methyl-coM	0.6368	0.712749	0
MF0120: lactate consumption	0.6606	0.732665	+
MF0010: tyrosine degradation (phenol pathway)	0.7093	0.779591	+
MF0053: allose degradation	0.7592	0.811826	+
MF0040: lysine degradation (cadaverine pathway)	0.7466	0.811826	-
MF0029: threonine degradation (glycine pathway)	0.7719	0.811826	+
MF0027: cysteine degradation	0.7719	0.811826	+
MF0020: valine degradation	0.7592	0.811826	-
MF0013: glutamate degradation (crotonyl-CoA pathway)	0.8359	0.871622	+
MF0069: galactarate degradation	0.9011	0.931646	+
MF0123: propionate production (propanediol pathway)	0.9274	0.95078	-
MF0068: glucarate degradation	0.9406	0.956277	-
MF0098: methanogenesis from carbon dioxide	0.9604	0.9604	+
MF0056: galactose degradation (Leloir pathway)	0.9538	0.9604	+
